# Role of Eyes and Eyes Protection Amidst SARS-CoV-2 Infection

**DOI:** 10.31729/jnma.5634

**Published:** 2021-01-31

**Authors:** Bibechan Thapa

**Affiliations:** 1 KIST Medical College and Teaching Hospital, Lalitpur, Nepal

**Keywords:** *eye protection*, *route of transmission*, *SARS-CoV-2*

## Abstract

Severe Acute Respiratory Syndrome Coronavirus 2 pandemic has infected millions of people. The conjunctival epithelium is easily exposed to infectious droplets and body fluids making eyes a potential route and reservoir of the infection. The CD147 and ACE2 receptor has been demonstrated in ocular surface cells, which implies that these cells may facilitate as a portal of entry for transmission of Severe Acute Respiratory Syndrome Coronavirus 2. Despite low viral load in tears and conjunctival swab, the negative RT-PCR results cannot exclude the possibility of the presence of Severe Acute Respiratory Syndrome Coronavirus 2 in ocular secretions. Pathogens might be transported by constant tear flow through the lacrimal duct system to the respiratory tract causing infection. Eyes are unlikely to be the main transmission route, however, their role in the transmission of Severe Acute Respiratory Syndrome Coronavirus 2 cannot be overlooked. Therefore, proper eye protection should be instituted while attending Severe Acute Respiratory Syndrome Coronavirus 2 positive individuals, especially by health professionals.

## INTRODUCTION

Since December 2019, the Severe Acute Respiratory Syndrome Coronavirus 2 (SARS-CoV-2) pandemic has infected and affected huge populations all around the world. The main route of transmission of SARS-CoV-2 seems to be airborne droplets, close contact with an infected person, or contact with infected fomites. The ocular transmission has also been reported with great concern^1^ which should not be underestimated.

## THE EYE IS A SUSCEPTIBLE ORGAN

Respiratory viruses cause numerous diseases in humans, including several ocular tropisms. The main reason for this is the easy contamination of conjunctival epithelium by infectious droplets and bodily fluids.^1^ Especially, hand-eye contacts may play an intrinsic role in transmitting the virus. The eyes may represent a source of transmission through infected tears as well as a window for infection via respiratory droplets and aerosolized particles coming in contact with the conjunctiva.^2^

## AN OCULAR MANIFESTATION OF SARS-COV-2

A study by Nan Hong et. Al.^3^ among patients with SARS-CoV-2 infection showed aggravated ocular symptoms in 27%, among which 11% had prodromal ocular symptoms.

Ocular symptomatology is relatively common and may present just before the onset of respiratory symptoms. The ocular manifestations such as follicular conjunctivitis have been repeatedly noted as an initial or subsequent symptom of SARS-CoV-2-positive patients.

In a systematic review^4^, the prevalence of ocular signs and symptoms varies from 0 to 31.58% while the detection rate of SARS-CoV-2 in the ocular sample ranged from 0 to 11.1%. Conjunctivitis was a relatively rare occurrence. The ocular manifestations were not always consistent with the detection of SARS-CoV-2 infection by reverse transcription-polymerase chain reaction (RT-PCR), done in the conjunctival swab samples and the opposite was also found to be true.

## ANATOMICAL IMPLICATIONS

The nasolacrimal system acts as an anatomical bridge between two systems: the respiratory and the ocular system. The natural mechanism of tear flow dynamics from the ocular surface to the inferior meatus via the nasolacrimal system might also facilitate the movement of virus particles from the ocular surface to the respiratory tract^5^, thereby potentially colonizing the respiratory tract. This implies the eyes to be the potential site of infection and gateway for the respiratory infection.

## MOLECULAR IMPLICATIONS

The structure of the cellular receptors and their distribution in respiratory and ocular systems are likely to aid the tissue tropism of most of the respiratory viruses, that now includes SARS-CoV-2, such as the host epithelial cell glycoproteins bearing terminal sialic acids which are thoroughly distributed all over the respiratory and ocular system. It indicates that ocular discharges containing viruses may come in contact with the respiratory tract through the nasolacrimal route and lead to infection manifesting as pneumonia.^6^ Previous studies point out CD147 as a transmembrane glycoprotein, a new spike protein (SP) receptor, and the interaction between CD147 and SP facilitates SARS-CoV-2 invasion of host cells.^7^ The presence of ACE2, another receptor revealed that ocular surfaces are susceptible to SARS-CoV-2 infections, and these cells may result in the transmi ssion of this virus to a human.^1^ Both CD147 and ACE2 have been demonstrated in ocular surfaces cells, these cells may serve as a portal for entry and reservoirs for the person to person transmission of respiratory virus like SARS-CoV-2.

## LOW VIRAL LOAD

The virus concentration in tears seems to be significantly less in comparison to the respiratory samples. Even PCR done on pharyngeal swab has an approximately 30% positive rate, thus making it more difficult to detect SARS-CoV-2 on the ocular samples. Also, the amount of sample that can be collected from the eye surface is naturally limited^8^ Significantly low detection of human-CoV-RNA by RT-PCR in conjunctival secretions from patients with SARS-CoV-2 may be related to the relatively low sensitivity of the current RT-PCR technique, later timing of sample collection, along with activation of the host immune system, significant increases in lactoferrin, secretory IgA levels in tears and increase in circulating IgM and IgG levels in plasma. Similar was demonstrated with SARS. Hence, negative RT-PCR results cannot exclude the possibilities of the presence of SARS-CoV-2 in ocular secretions.^9^

## THE EYES AS GATEWAY TO THE RESPIRATORY SYSTEM

Considering the vulnerability of the conjunctiva to the infectious droplets and fomite transmission, anatomical and physiological connection between mucosa of the upper respiratory tract and ocular surface and presence of same entry receptor in both respiratory and ocular surface for some respiratory viruses including SARS-CoV-2, the role of eyes in human coronavirus infection is crucial and cannot be overlooked.^9^ Studies suggest the eye is less often involved in SARS-CoV, Middle East Respiratory Syndrome-CoV (MERS-CoV), and 2019-nCoV infection. Even conjunctivitis has been less reported in SARS-CoV-2 and never reported in SARS and MERS patients.^4,9^ This hints that the eye is neither a preferred organ nor is a suitable entry point for human CoV infection that enables the virus to colonize and infect the respiratory tract. However, the pathogens that come in contact with the ocular surface might be transported by regular drainage of a tear through the lacrimal duct system to reach the respiratory tract and cause infection.^9^ Some studies also have shown ocular symptoms to be relatively common in SARS-CoV-2.^2,3^ Whilst the eye is unlikely to be the main transmission route, its role in the transmission of SARS-CoV-2 cannot be excluded.^4^

## NECESSITIES OF EYE PROTECTION

Considering the high viral aerosol load in the hospital, the ocular transmission route should be seriously considered. A breach in eye protection may lead to exposure of the ocular surface to potentially infectious agents like virus, which may then transmit to the hands through eye-hand contact and eventually to other mucous membranes like the mouth. Therefore, along with regular masks, gowns, and gloves; goggles, and frequent hand washing, especially for medical personals who are in close proximity with SARS-CoV-2 patients is utmost.^1^ One of the risk factors for the nosocomial infection of medical personals with SARS was whether they wore protective goggles. Only one percent of the clinicians wearing eye protection were infected compared to eight percent of the clinicians infected who did not wear eye protection.^10^ Similar is true in SARS-CoV-2 infection^3^ thus optimal use of eye safety measures is mandatory.

Ocular symptoms are found to be relatively common and viruses have also been demonstrated particularly in those with ocular symptoms with SARS-CoV-2. Therefore, there is a possibility of the virus transmitting from the ocular surface. All health workers should be vigilant about ocular symptoms consistent with SARS-CoV-2 infection, and optimally use eye protection such as goggles or face shields as an absolute part of the standard personal protective equipment, and should consider tears, ocular discharges, and ocular contaminants to be potentially infectious.^2^

## CONCLUSIONS

Based on the anatomical, molecular, and current clinical evidence, the possibility of the ocular surface being an infection gateway is low but the role of the eyes in the transmission of SARS-CoV-2 infection cannot be overlooked and ought not to be underestimated. SARS-CoV-2 infection or transmission through the ocular surface may be a potential infection route, especially in hospitals where infected aerosols are frequent. Therefore, appropriate ocular protection is very important to prevent infection, especially for medical personnel.
